# Dexmedetomidine nasal administration improves perioperative sleep quality and neurocognitive deficits in elderly patients undergoing general anesthesia

**DOI:** 10.1186/s12871-024-02417-9

**Published:** 2024-01-30

**Authors:** Jiang He, Xinning Zhang, Cuicui Li, Baojun Fu, Yizhou Huang, Heng Li

**Affiliations:** 1 Department of Anesthesiology, The Sixth Affiliated Hospital of Guangzhou Medical University, Qingyuan, China; 2 Department of Gynaecology, The Sixth Affiliated Hospital of Guangzhou Medical University, Qingyuan, China

**Keywords:** Dexmedetomidine, Nasal administration, General anesthesia in elderly patients, Perioperative sleep quality, Neurocognitive impairment

## Abstract

**Objective:**

To investigate the improvement of perioperative sleep quality and neurocognitive impairment in elderly patients under general anesthesia by nasal administration of dexmedetomidine.

**Methods:**

One hundred and twenty patients admitted to our hospital for various laparoscopic elective gynecological surgeries lasting more than 1 h under general anesthesia from July 2021 to March 2023 were selected. All subjects were divided into 3 groups according to the random number table method. From 21:00 to 21:30 every night from one day before to 5 days after surgery, group A was given alprazolam 0.4 mg orally; group B was given dexmedetomidine 1.5ug/kg nasal drip; group C was given saline nasal drip. All subjects were observed for general information, sleep quality, postoperative cognitive function, anxiety status, sleep quality, adverse effects and complication occurrence.

**Results:**

The difference in general information between the three groups was not statistically significant, *P* > 0.05; the sleep quality scores of the three groups on admission were not statistically significant, *P* > 0.05. At the Preoperative 1d, postoperative 1d, 3d and 5d, the RCSQ scores of the subjects in group A and group B were higher than those in groups C, and with the postoperative RCSQ scores of subjects in group B were higher as the time increased; the assessment of anxiety status in the three groups 1d before surgery was not statistically significant, *P* > 0.05. The cognitive function scores of subjects in the three groups were not statistically significant in the preoperative 1d, *P* > 0.05. The postoperative 1d (24.63 ± 2.23), 3d (25.83 ± 2.53), and 5d (26.15 ± 2.01) scores of the subjects in group B were higher than those in groups A and C (*P* < 0.05), and the subjects in group B had better recovery of postoperative cognitive function with increasing time; the occurrence of postoperative delirium (POD) in group B (12.5%) were lower on postoperative 5d than those in groups A (37.5%) and C (32.5%) (*P* < 0.05). There was no statistical significance in the evaluation of anxiety state of the three groups on the first day before operation (*P* > 0.05). The scores in group B were lower than those in group C on the postoperative 1d, 3d, 5 d (*P* < 0.05). The overall incidence of adverse reactions and complications in subjects in group B was 17.5% significantly lower than that in groups A and C (*P* < 0.05).

**Conclusion:**

Dexmedetomidine can effectively improve the sleep disorder of elderly general anesthesia patients, reduce the damage to their neurocognitive function and the occurrence of POD, effectively reduce the anxiety of patients and the occurrence of adverse reactions and complications, and has better sedative, improve postoperative cognitive function and anti-anxiety effects, with a high drug safety, worthy of clinical application and promotion.

## Introduction

With the advent of aging society, more and more elderly patients need to undergo surgery under general anesthesia to treat their diseases [1]. According to clinical literature [2], factors such as anesthesia time, anesthesia level, and anesthetic drugs can have different degrees of influence on patients, and there is also a certain correlation between these factors, which can rapidly eliminate anesthetic drugs from the organism after surgery, accelerate the postoperative recovery of patients, and reduce the occurrence of various side effects. Currently, along with the increase in the elderly population, the number of elderly patients under general anesthesia is growing, and at the same time, the importance of the effects on the brain function of elderly patients after general anesthesia is increasing [3]. In 2018, experts from multiple specialties formed the Perioperative Cognitive Nomenclature Working Group to describe “Periop-erative neurocognitive disorders (PND)” in patients undergoing general anesthesia [4] Perioperative neurocognitive disorders consist of two main aspects: postoperative delirium (POD) and postoperative cognitive dyfunction (POCD) [5]. Although there are many similarities between postoperative delirium and postoperative cognitive dysfunction, and they both present clinically with symptoms of reduced or diminished cognitive function, these two disorders have many differences in terms of theories, time of onset, and diagnostic criteria [6]. Previous studies have found that melatonin, ramelteon and esketamine have certain therapeutic effects on POD and POCD, but in the study of Aiello G [7], melatonin and Ramtern did not seem to reduce the psychosis of patients in intensive care unit. Right, in the study of Ma J [8], it was found that low-dose esectamine can improve neurocognitive dysfunction in elderly patients with gastrointestinal tumors after general anesthesia, which can be obtained from the above studies, and the research results are not uniform. Perioperative neurocognitive dysfunction often leads to increased hospital stays and increases the financial stress of patients, further reducing their overall quality of life. To date, a large number of studies have discussed how factors associated with general anesthesia affect patients’ cognitive function [9]. However, the exact mechanism of action is not clear, and therefore the results obtained are varied [10]. Older patients are declining in all aspects of body function, the weight of the brain is decreasing, and neuronal and cerebral blood flow is decreasing, which makes them more sensitive to general anesthetic drugs [11]. In addition, general anesthesia also affects the sleep quality of elderly patients to some extent. As the brain tissues of elderly patients show different degrees of degeneration, the oxygenation of the organism also has a significant decrease, and they are more prone to sleep problems after surgery, which can have a greater impact on the postoperative recovery of patients [12]. Dexmedetomidine is an a2 agonist in clinical practice, and when it enters the patient’s organism, it has an inhibitory effect on the patient’s central nervous system conduction, and, at the same time, it also has an effect on the nerve reflexes and reduces the patient’s pain level [13]. Therefore, in this study, 120 patients admitted to our hospital for various elective laparoscopic gynecological surgeries lasting more than 1 h under general anesthesia from July 2021 to March 2023 were selected, and all subjects were divided into 3 groups according to the randomized numerical table method, and their relevant studies were conducted, and the results of the study are reported below.

## Information and methods

### General data

**Fig. 1:**
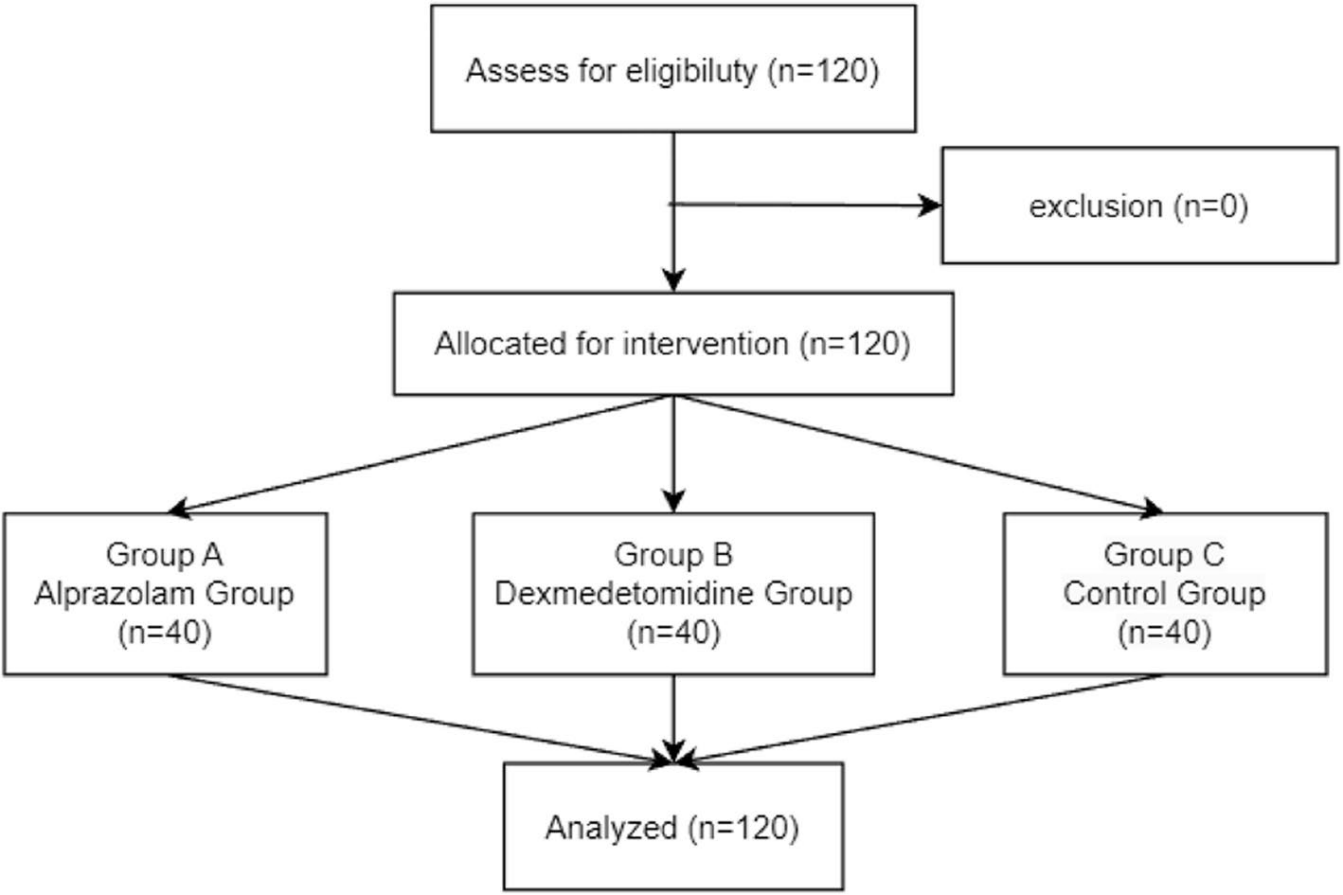
Inclusion procedure of the participants

One hundred and twenty patients admitted to our hospital for various elective laparoscopic gynecological surgeries lasting more than 1 h under general anesthesia from July 2021 to March 2023 were selected. On admission all subjects were divided into 3 groups according to the random number table method: alprazolam group (group A, *n* = 40), dexmedetomidine group (group B, *n* = 40) and control group (group C, *n* = 40). The drugs were all configured by third-party personnel in the experimental group, with uniform syringes and volumes, and numbered, and administered by the dosing operator physician according to the established patient drug number. Upon admission, patients’ sleep status before admission was assessed using the Pittsburgh Sleep Quality Index scale, and basic patient information was recorded: age, ASA classification, and BMI. The consort diagram were performed in Fig. 1.

Sample size calculation formula [14]:

$$n_1=n_2=2\left[\frac{\left(u_\alpha+u_\beta\right)}{\displaystyle\frac\delta\sigma}\right]^2+\frac14u_\alpha^2$$

δ/σ = 0.88, test level α = 0.05(unilateral), test efficacy β = 0.10, calculated n1 = n2 = 48, considering 20% of the lost follow-up rate, the final calculated n1 = n2 = 60 cases, a total of 120 cases.

The study was reviewed and approved by the ethics committee (Reg.NO.IRB-2020–087). Subjects and family members signed the informed consent form.

Inclusion criteria: ① American Society of Anesthesiologists (ASA) physical status Classes II and III; ② laparoscopic gynecological surgery; ③ patients aged 65–80 years; ④ The patient had no previous medical history.

Exclusion criteria: ① history of cardiac insufficiency or echocardiography showing the left ventricular ejection fraction (LVEF) < 55%; ② any cerebrovascular event within 3 months, such as stroke, transient ischemic attack (TIA), etc.; ③ patients with any mental illness; ④ patients abusing narcotic analgesic sedative drugs; ⑤ patients taking sedative hypnotic drugs for a long time; ⑥ patients with previous allergic rhinitis or nasal surgery.

### Methods

#### Perioperative management

Patients were given alprazolam 0.4 mg orally from 21:00 to 21:30 every night from one day before to 5 days after surgery in group A; dexmedetomidine 1.5ug/kg nasal drip was given in group B, the drug concentration was 100ug/ml, the syringe size was 1ml, the left and right nostrils were administered alternately, 0.2ml/time in each nostril, and the interval between administration in the same nostril was 5min until the administration was completed; group C was given Group C was given physiological saline nasal drops.

During the administration period, vital signs were routinely monitored and appropriate treatment was given by the surgeon on duty in case of hypertension, hypotension, tachycardia, bradycardia and respiratory depression. Monitoring and medical records were checked from 8:00 to 8:30 the next morning to record the occurrence of the above-mentioned adverse reactions.

#### Homogenization of perioperative management

The entire perioperative management measures were executed under the appropriate application of the ERAS concept and the consistency of the execution protocol between the two groups was ensured.

Fasting and abstinence from food and drink for 8 h prior to surgery. After admission, the upper limb veins were opened and the ECG, noninvasive blood pressure and pulse oximetry, partial pressure of end-expiratory carbon dioxide and EEG dual spectrum index were routinely monitored. General anesthesia was induced with etomidate 0.2mg·kg-1, propofol 1mg·kg-1, sufentanil 0.4μg·kg-1, and cis-atracurium 0.2 mg·kg-1, tracheal intubation was performed after the onset of inotropy. Ventilation was performed using a lung-protective ventilation strategy: low tidal volume (6 ml/kg); inspiration-exhalation ratio of 1:1.5–2.0; intraoperative adjustment of ventilation frequency to maintain the partial pressure of end-expiratory carbon dioxide (PETCO2) at 35–45 mmHg. Maintenance of anesthesia: propofol 6–10 mg·kg-1·h-1, intravenous pumping of remifentanil 0.1–0.2μg·kg-1·min-1 and cis-atracurium 1 μg·kg-1·min-1 were used to maintain the BIS at 40–60. Both cis-atracurium and propofol were discontinued when the laparoscope was stopped to close the peritoneum. Intraoperatively, a goal-directed fluid therapy concept was applied to guide the infusion of fluids, and balanced fluids were applied to maintain the inlet and outlet balance, with crystals 3:1 colloid. Intraoperative fluid volume changes were minimized to maintain intraoperative MAP fluctuations of no more than 20% of basal values. Abdominal transverse fascial block analgesia was performed at the end of surgery, and postoperative VAS score was controlled more than 3 using multimodal analgesic mode, with slow intravenous flurbiprofen ester 50 mg of non-steroidal anti-inflammatory drug if necessary. In our research, we designed to use opioids or tramadol combined with NSAIDs to control the postoperative VAS score below 3.

### Treatment of adverse reactions

Subjects with any adverse reactions in the treatment process need to be treated symptomatically in a timely manner and treated with the appropriate drugs if necessary.

### Observation content

All subjects were observed for general information, sleep quality, postoperative cognitive function, anxiety status, adverse reactions and occurrence of complications.

### Evaluation criteria

1. The PSIQ was used at admission to evaluate the patient’s sleep status in the last month prior to admission. the PSQI was used to rate the subject’s sleep quality in the last 1 month, with a total score ranging from 0–21, with higher scores indicating poorer sleep quality. The Richards–Campbell Sleep Questionnaire [15] (RCSQ) was used to record the patients’ sleep quality star before surgery and at 1, 3 and 5 days after surgery. The counting method was recorded with numbers (0 points to 100 points). The higher the score, the higher the sleep quality star.
2. Cognitive function was tested by Mini-Mental State Examinon (MMSE) and confusion assessment method (CAM) before surgery and at 1, 3 and 5 days after surgery [16]. The MMSE is the most commonly used cognitive screening scale in the world and provides simple ratings of orientation, memory, language, computation, and attention. With a total score of 30, a higher score indicates a lower incidence of neurocognitive dysfunction. CAM includes four aspects: (1) acute onset and fluctuating course, (2) inattention, (3) incoherent thinking, and (4) altered consciousness; the presence of both (1) and (2), and one of (3) or (4) is diagnostic of POD.
3. The Generalized Anxiety Disorder Scale (GAD-7) [17] was used to assess anxiety before surgery and at the 1st, 3rd, 5th day after surgery. GAD-7 has good reliability and validity. Due to its advantages of simplicity and reliability, it has been widely used in clinical screening for anxiety disorders in recent years. There are 7 items in total, with 3 points for each item. The total score of the 7 items is added together, and the score ≥ 5 is classified as anxiety.
4. The incidence of adverse reactions and complications, such as hypertension, tachycardia, pulmonary infection and postoperative bleeding, etc. in the three groups of subjects during treatment were analyzed.

### Statistical methods

SPSS26.0 software was applied for statistical analysis. Normally distributed measurement data were expressed as mean ± standard deviation, independent sample t-test was used for comparison between two groups, count data were analyzed by chi-square test or Fisher’s exact probability method, non-normally distributed data and ordered categorical data were analyzed by rank sum test, and differences were considered statistically significant at *P* < 0.05.

## Results

### General information

The difference between the general information of the three groups of subjects was not statistically significant, *P* > 0.05. Table 1 for more details.

**Table 1:** Comparison of general information of subjects in the three groups

Group	Number	Age (years)	BMI (kg/m^2^)	ASA (II/III)	PSQI
Group A	40	68.50 ± 2.93	21.79 ± 2.36	20/20	6.75 ± 1.96
Group B	40	68.45 ± 2.82	21.76 ± 1.59	22/18	5.93 ± 2.29
Group C	40	69.20 ± 3.01	22.45 ± 2.09	21/19	6.63 ± 2.38
F/x^2^	-	0.824	1.464	0.643	0.942
*P*	-	0.441	0.236	0.725	0.393

### Sleep quality score

**Fig. 2:**
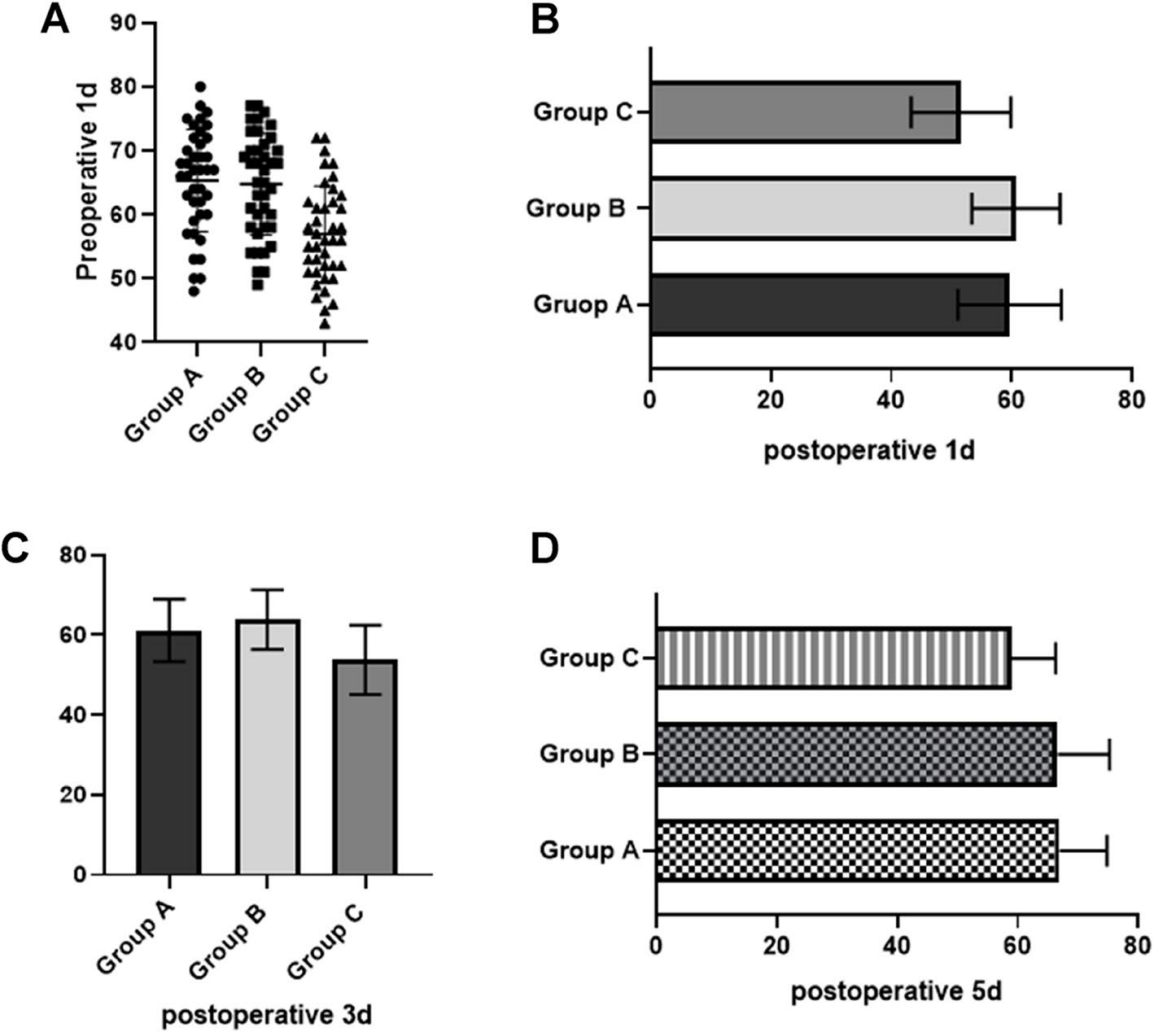
Sleep quality scores of subjects in the three groups (**A** Comparison of preoperative sleep quality scores among the three groups; **B** Comparison of sleep quality scores among the three groups one day after surgery; **C** Comparison of sleep quality scores among the three groups three days after surgery; **D** Comparison of sleep quality scores among the three groups five days after surgery)

Based on the RCSQ results, the preoperative 1d, postoperative 1d, 3d, and 5d sleep quality scores of the subjects in group A and B were higher than those in groups C, and the postoperative sleep quality scores of the subjects in group B were higher with increasing time, *F* = 14.247,15.451, 15.841, 12.848, *P* = 0.000. Table 2 and Fig. 2 for more details.

**Table 2:** Sleep quality scores of subjects in the three groups

Group	Number	Preoperative 1d	Postoperative 1d	Postoperative 3d	Postoperative 5d
Group A	40	65.32 ± 8.01	59.68 ± 8.57	61.15 ± 7.79	66.95 ± 7.85
Group B	40	64.77 ± 7.94	60.75 ± 7.33	63.83 ± 7.46	66.62 ± 8.63
Group C	40	56.97 ± 7.51	51.58 ± 8.26	53.78 ± 8.67	58.97 ± 7.36
*P*	-	< 0.0001	< 0.0001	< 0.0001	< 0.0001

### Postoperative cognitive function measurement

**Fig. 3:**
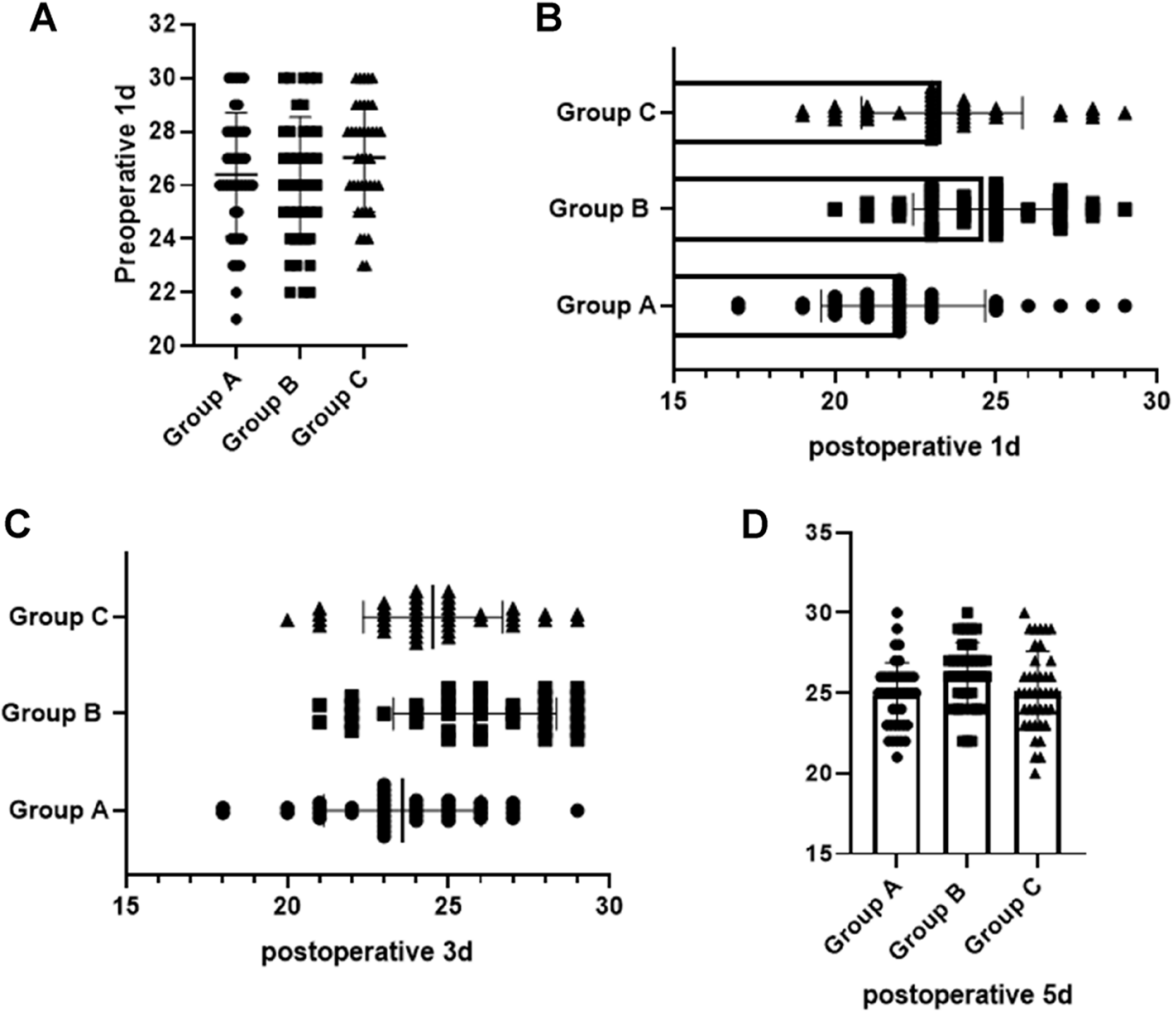
Postoperative cognitive function measurements in the three groups of subjects. (**A** Comparison of preoperative cognitive function measurements among the three groups; **B** Comparison of cognitive function measurements among the three groups one day after surgery; **C** Comparison of cognitive function measurements among the three groups three days after surgery; **D** Comparison of cognitive function measurements among the three groups five days after surgery)

The preoperative 1d cognitive function scores of the subjects in the three groups were not statistically significant, *P* > 0.05. The postoperative 1d (24.64 ± 2.23), 3d (25.83 ± 2.53), and 5d (26.15 ± 2.01) scores of the subjects in group B were higher than those in groups A and C, and the subjects in group B had better recovery of postoperative cognitive function with increasing time, F = 29.116, 28.200, 30.228, *P* = 0.000. Table 3 and Fig. 3 for more details.

**Table 3:** Postoperative cognitive function measurements in the three groups of subjects

Group	Number of cases	Preoperative 1d	Postoperative 1d	Postoperative 3d	Postoperative 5d
Group A	40	26.45 ± 2.41	22.13 ± 2.54	23.58 ± 2.45	24.87 ± 2.45
Group B	40	26.25 ± 2.52	24.63 ± 2.23	25.83 ± 2.53	26.15 ± 2.01
Group C	40	27.13 ± 2.15	23.40 ± 2.68	24.65 ± 2.49	25.27 ± 2.69
*p*	-	0.226	< 0.0001	< 0.0001	< 0.0001

### POD incidence

There was no statistically significant difference in the incidence of POD between the three groups of patients at postoperative 1d, 3d, and the incidence of POD was lower in group B than in groups A and C at postoperative 5d (*P* < 0.05). Table 4 for more details.

**Table 4:** Occurrence of POD in the three groups of subjects

Group	Number of cases	Postoperative 1d	Postoperative 3d	Postoperative 5d
Group A	40	3 (7.5)	12 (30)	15 (37.5)
Group B	40	1 (2.5)	5 (12.5)	5 (12.5)
Group C	40	4 (10)	11 (27.5)	13 (32.5)
*P*	-	0.348	0.135	0.036

### Anxiety state assessment

**Fig. 4:**
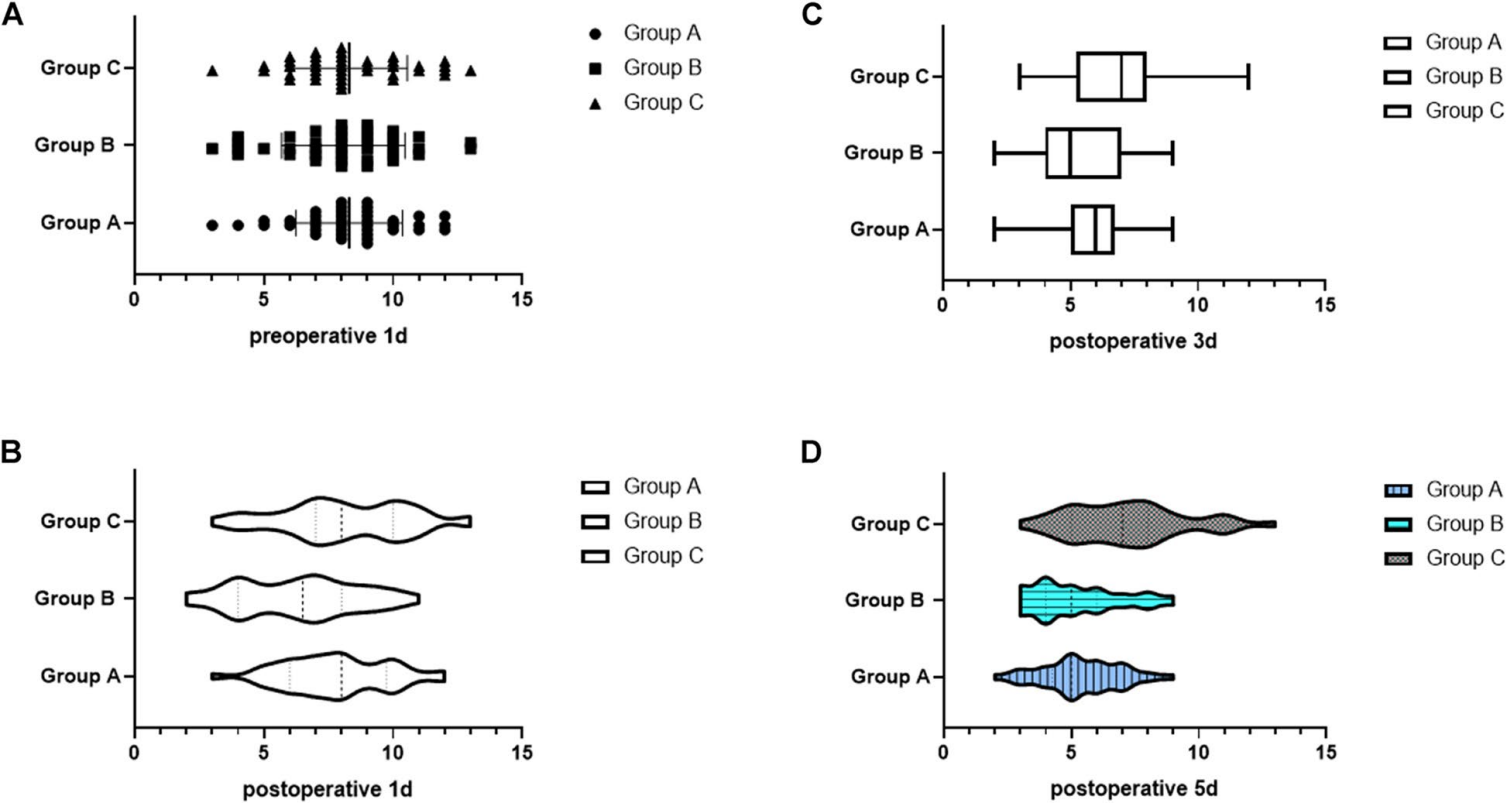
Anxiety state assessment of the three groups of subjects (**A** Comparison of preoperative anxiety state assessment among the three groups; **B** Comparison of anxiety state assessment among the three groups one day after surgery; **C** Comparison of anxiety state assessment among the three groups three days after surgery; **D** Comparison of anxiety state assessment among the three groups five days after surgery)

The anxiety state assessment of the subjects in the three groups at preoperative 1 d was not statistically significant, *P* > 0.05. Subjects in group B had lower scores at 1d postoperatively (6.30 ± 2.35), 3d postoperatively (5.50 ± 1.60), 5d postoperatively (5.17 ± 1.78) than those in group C, and along with the extension of time, the lower the postoperative anxiety state scores of subjects in group B. The difference between the three groups was statistically significant, *P* < 0.05. Table 5 and Fig. 4 for more details.

**Table 5:** Anxiety state assessment of the three groups of subjects

Group	Number of cases	Preoperative 1d	Postoperative 1d	Postoperative 3d	Postoperative 5d
Group A	40	8.30±2.07	7.80±2.04	5.65±1.73	5.40±1.55
Group B	40	8.08±2.39	6.30±2.35	5.50±1.60	5.17±1.78
Group C	40	8.30±2.24	8.13±2.46	6.93±1.86	7.18±2.29
F	-	0.135	7.233	8.248	13.337
*p*	-	0.874	0.001	0.000	0.000

### Occurrence of adverse reactions and complications

**Fig. 5:**
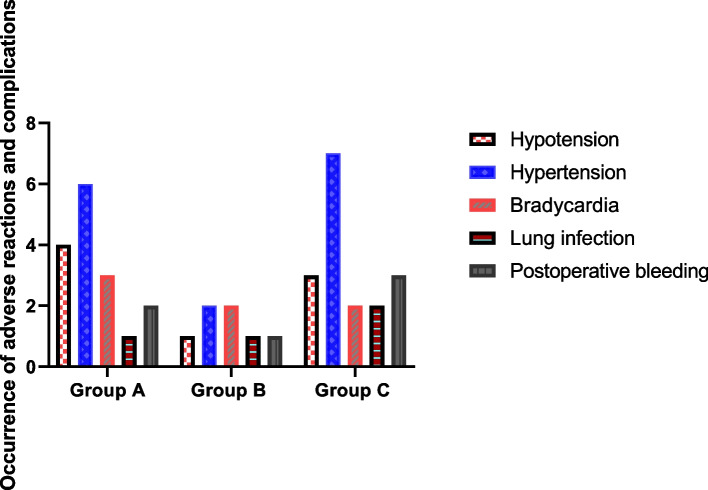
Occurrence of adverse reactions and complications in the three groups of subjects

No respiratory depression occurred in the three groups, the overall incidence of adverse reactions and complications in subjects in group B was 17.5% significantly lower than in groups A and C, X^2^ = 6.825,*P* = 0.033. Table 6 and Fig. 5 for more details.

**Table 6:** Occurrence of adverse reactions and complications in the three groups of subjects [(n)%]

Group	Number	Hypotension	Hypertension	Bradycardia	Lung infection	Postoperative bleeding	Total incidence
Group A	40	4 (10.00)	6(15.00)	3 (7.50)	1 (2.50)	2 (5.00)	16 (40.00)
Group B	40	1 (2.50)	2(5.00)	2 (5.0)	1 (2.50)	1 (2.50)	7 (17.50)
Group C	40	3 (7.50)	7(17.50)	2 (5.00)	2 (5.00)	3 (7.50)	17 (42.50)
X^2^	-						6.825
*p*	-						0.033

## Discussion

General anesthesia is the most common form of anesthesia in surgical procedures, and it is used most frequently in patients in the coming years [18]. With the increasing age of the elderly population, there is a significant decrease in immune capacity and resistance in their body functions, as well as disorders in systemic physiological functions, so elderly patients are more prone to more intense stress reactions during surgical treatment, so it is crucial to choose the appropriate anesthetic for surgery [19]. Clinically relevant studies have shown [20] that cognitive dysfunction is the most common complication in elderly patients undergoing general anesthesia after surgery, which on the one hand can prolong the overall hospital stay and on the other hand can lead to different degrees of impairment of the patient's neurological and endocrine systems and other primaries, which in turn can have a certain impact on postoperative recovery. Perioperative sleep dysfunction is the most common clinical manifestation of postoperative brain dysfunction in patients, which is mainly correlated with the use of anesthetic drugs, living environment, body fever, and duration of surgery, and can lead to hemodynamic disorders, sleep dysfunction, and postoperative sleepiness, and more severe patients may experience negative emotions such as depression and anxiety [21]. Because of the severe degeneration of both psychological and physiological functions in elderly patients, the problem of sleep disturbance arises more severely compared to younger people. General anesthesia has a strong central inhibitory effect on patients, who have a low level of consciousness, but it also has a certain impact on their psychology and stimulates the development of sleep disorders [22]. Dexmedetomidine is one of the a2-adrenergic receptor agonists, and its high selectivity and efficiency are its main characteristics, and it is used most frequently in patients under general anesthesia, choosing a2-adrenergic receptors, which not only have a direct effect on the external nerves, but also can effectively inhibit the secretion of metanephrine in the patient’s organism [23]. In addition, it can effectively inhibit pain and accelerate the secretion of acetylcholine in the organism, which makes the synthesis of nitric oxide (NO) faster, improves the effect of sedation and analgesia, and effectively regulates the negative emotions of patients [24].

The results of the current study showed that subjects in group A and B had higher perioperative period sleep quality scores than those in groups C, and with the extension of time, subjects in group A and B had higher postoperative sleep quality scores, indicating that dexmedetomidine can effectively alleviate patients’ postoperative sleep disorders and significantly improve patients’ sleep function, action comparable to alprazolam. The main reason for this is that dexmedetomidine can effectively regulate the activity function of the patient’s noradrenergic system to ensure natural non-rapid eye movement sleep, and at the same time, effectively improve the patient’s hyperactive cerebral cortex, which makes the patient’s disordered sleep function improve, and the hypnotic effect produced by the sedative function of dexmedetomidine has a high similarity to natural sleep [25].

The anesthetic drugs used by patients prior to general anesthetic surgical treatment accelerate the metabolic rate of brain tissue and cause a high degree of damage to functional cells in the brain nerves, which can cause cognitive dysfunction and aggravate brain nerve tissue damage in patients after surgery [26]. The results of the present study showed that subjects in group B had higher postoperative scores at 1 d, 3 d, 5 d, and lower occurrence of POD at 5d than those in groups A and C, point out the subjects in group B had better recovery of postoperative cognitive function with longer time. This indicates that dexmedetomidine can significantly improve the postoperative cognitive function, reduce the degree of brain tissue damage in elderly general anesthesia patients. Studies have shown that preoperative sleep disturbances increase the incidence of POD and prolong the length of stay in elderly patients [27]. In the present study, the quality of sleep was significantly improved in group A after administration of alprazolam compared with the control group, but there was no significant improvement in postoperative MMSE scores and POD incidence, which may be related to the short duration of sleep improvement and the possibility that benzodiazepines themselves may cause delirium. In contrast, dexmedetomidine could reduce the incidence of POD. It suggests that dexmedetomidine can improve the cognitive function of the elderly through other mechanisms besides improving sleep. The main reason for this is that dexmedetomidine can exert a protective effect on the nervous system by regulating the amount of protective and damaging cytokine production and blocking or restoring the signaling pathways associated with the nervous system [28], Consistent with the findings of Skrobik Y [29] et al.

The results of the current study showed that subjects in group B had lower the anxiety state scores at postoperative 1 d, 3 d, 5 d than those in groups C. With the increase in time, subjects in group B had lower postoperative the lower the anxiety state score was, the greater the difference between the three groups was statistically significant (*P* < 0.05), showing that dexmedetomidine could effectively reduce the anxiety of elderly general anesthesia patients and reduce the generation of adverse emotions. Meanwhile, the overall incidence of adverse reactions and complications in subjects in group B was 17.5% significantly lower than that in groups A and C, indicating that dexmedetomidine can significantly reduce the incidence of adverse reactions and complications in elderly patients under general anesthesia. Although dexmedetomidine has a sympathetic nerve depressant effect on the patient’s body, which may cause a decrease in blood pressure and bradycardia, in this study, the number of cases of hypertension and hypotension in Group B decreased compared to Group A and Group C, and bradycardia does not increase. The reason may be related to the drug action and administration method of dexmedetomidine. On the one hand, dexmedetomidine has a certain analgesic effect, reducing pain induced hypertension in patients; On the other hand, dexmedetomidine administered through the nasal cavity has a slow onset rate and avoids adverse reactions such as blood pressure drop and bradycardia caused by intravenous injection [30]. Dexmedetomidine has a better postoperative sedative and analgesic effect.

In conclusion, dexmedetomidine can effectively improve the sleep disorder of elderly patients under general anesthesia, reduce the damage to their neurocognitive function, effectively reduce patients’ anxiety, reduce the occurrence of adverse reactions and complications, and has better sedative, analgesic and anti-anxiety effects, while the total amount of drugs used intraoperatively and postoperatively is less and has a high medication safety, which is worthy of clinical application and promotion.

However, there are still shortcomings in this study. For example, the sample size in this study is small, so the current study has not been fully analyzed, so it is not universal. In the follow-up study, the number of cases should be increased for in-depth study to ensure the accuracy of research data. Meanwhile, we used intravenous sodium chloride in control subjects, which may somehow influence the outcomes due to the different chemical preparations. Further research were needed to eliminate the possible influence.

## Data Availability

The experimental data used to support the findings of this study are available from the corresponding author upon request.
